# Sugarcane-Based Polyethylene Biocomposite Reinforced with Organophilic Montmorillonite Clay: Experimental Characterization and Performance Evaluation

**DOI:** 10.3390/polym16223215

**Published:** 2024-11-20

**Authors:** Gustavo H. A. Barbalho, José J. S. Nascimento, Lucineide B. Silva, João M. P. Q. Delgado, Jackson B. Simões, Vital A. B. Oliveira, Luis E. A. Santos, Maria J. Figueiredo, Francisco S. Chaves, Antonio G. B. Lima

**Affiliations:** 1 Federal Institute of Education, Science and Technology of Rio Grande do Norte, Canguaretama 58190-000, Rio Grande do Norte, Brazil; gustavo.barbalho@ifrn.edu.br; 2 Department of Materials Engineering, Federal University of Campina Grande, Campina Grande 58429-900, Paraiba, Brazil; jeffpesquisador@gmail.com; 3 Department of Materials Engineering, Federal University of Paraiba, João Pessoa 58051-900, Paraiba, Brazil; lucineide@ct.ufpb.br; 4 CONSTRUCT-LFC, Department of Civil Engineering, Faculty of Engineering, University of Porto, 4200-465 Porto, Portugal; 5 Engineering Department, Rural Federal University of Semi-Arid (UFERSA), Caraúbas 59780-000, Rio Grande do Norte, Brazil; jackson.simoes@ufersa.edu.br; 6 Department of Education, State University of Paraiba, Guarabira 58200-000, Paraíba, Brazil; profvitaloliveira@gmail.com; 7 Postgraduate Program in Process Engineering, Federal University of Campina Grande, Campina Grande 58429-900, Paraíba, Brazil; santomorenynho@gmail.com; 8 Department of Agro-Industrial Management and Technology, Federal University of Paraíba, Bananeiras 58220-000, Paraiba, Brazil; mariaufp@gmail.com; 9 Department of Mechanical Engineering, Federal University of Campina Grande, Campina Grande 58429-900, Paraiba, Brazil; samuelchaves28@gmail.com (F.S.C.); antonio.gilson@ufcg.edu.br (A.G.B.L.)

**Keywords:** biopolymers, organophilic montmorillonite, compatibility, mechanical properties

## Abstract

With the growing human awareness of trying to reduce the environmental impact in today’s world, the development of new sustainably based materials has been the increasing focus of industry and academia. Biocomposites are environmentally friendly materials produced from raw materials synthesized from renewable sources. In this sense, this work aims to characterize and evaluate the mechanical and thermal performances of biocomposites manufactured from a thermoplastic matrix of high-density bioethylene and obtained from ethanol produced from sugarcane and reinforced with organophilic montmorillonite clay. For this, polyethylene grafted with maleic anhydride (PE-g-MA) was used as a compatibilizer. Dry biocomposites with 1, 3, and 5% organophilic montmorillonite clay, by weight, were subjected to structural (FTIR and DRX), thermal (DSC), thermogravimetric (TG/DTG), thermodynamic–mechanical (DMA), morphological (SEM and MET), and mechanical (tensile, flexural, impact, and shore D hardness tests) characterizations. The DMA experiments were carried out within the viscoelastic region of the polymer. From the obtained results, we notice that, in general, there was an increase in the properties of high-density biopolyethylene (B-HDPE) (without compromising its processability), and therefore, the automotive application of biocomposites compatible with PE-g-MA, containing low levels of organophilic montmorillonite clay, is recommended.

## 1. Introduction

The unfavorable environmental impacts provoked by the excessive use of non-biodegradable and non-renewable products originating from petroleum have stimulated research focused on the use of biodegradable material resources, such as bioplastics, for example. In the present day, bioplastics have become an important part of the bioeconomy, being responsible for an innovative and fast-growing industry that presents great potential to decouple economic growth from resource depletion and environmental impact. The degradation of bioplastics in different environments has been evaluated as being mainly dependent upon the composition of bioplastics and the environmental conditions. Incomplete degradation during waste management processes and the leakage of bioplastics into the environment are becoming major concerns that need to be further investigated. Unfortunately, the size of the bioplastics industry is still smaller and relatively new compared to the conventional plastics industry. However, regulations on the use and manufacturing of bioplastics have been improved in recent years. Therefore, it is understandable that several present and future challenges related to the adoption and use of bioplastics must be identified.

Biopolymers and related technological processes have been extensively evaluated. These materials are well-known for their environmental benefits. Thus, they are considered promising alternatives to petroleum-based polymers, as most biopolymers help reduce environmental pollution and CO_2_ gas emissions and tend to minimize strong greenhouse effects [1]. The research related to natural biocomposites has opened up great opportunities for the safe use of new high-performance raw materials. Materials such as green biocomposites (based on natural fibers and inorganic materials, for example) can now replace conventional non-renewable materials (based on fossil fuel sources, for example) [2]. In the present day, different synthetic polymers are manufactured from non-renewable petrochemical sources. However, environmental concerns and the use of polymers obtained from renewable sources have been greatly encouraged.

Biopolyethylene (B-HDPE), well-known as “green polyethylene”, is originally obtained from the ethylene produced by dehydration of ethanol, which is produced by the fermentation and distillation of sugarcane juice [3]. “Green polyethylene” has a similar structure to polymers originating from the petrochemical route, but it is a technological innovation in the manufacture of plastic products. Green polyethylene absorbs CO_2_ from the atmosphere during the production cycle, thus reducing the environmental impacts originating from raw materials of fossil fuel origin [4].

In recent decades, an alternative chemistry, called “green chemistry”, has emerged. The aim of the idea is to contribute to the reduction of human impacts on the environment. Polymers are involved in this trend, and various plastic materials from renewable sources are now used in a wide range of applications, such as packaging, leisure, agriculture, and biomedicine. Some of these polymers, so-called biotechnological polymers, such as polylactides (PLA), polyhydroxyalkanoates (PHA), and polysaccharides, combine excellent characteristics of renewability and biodegradability. From the commercial point of view, for these biodegradable polymers to be truly competitive with conventional petroleum-based plastics, their mechanical and thermal properties and moisture sensitivity must be improved significantly [5]. The use of biopolymers produced from renewable sources of raw material, such as corn, sugarcane, cellulose, and chitin, among others, remains an alternative, and “green” polymers, such as B-HDPE, although not biodegradable, can be produced without impacting the balance of carbon dioxide (CO_2_) in nature. Carbon dioxide is captured by sugarcane biomass from the atmosphere, and for the next step after harvest, it is released into the atmosphere through the combustion of the biomass. In sequence, it is then captured again by the growth of sugarcane biomass during the photosynthesis process before the next harvest [6].

As already mentioned, biopolymers present some advantages. However, certain technical limitations related to its processability and use as a final product can be cited as examples, namely the nature of the matrix and its interaction (adhesion) with the reinforcement. Thus, studies dedicated to evaluating the modifications of biopolymers through the use of additives have been conducted. These studies focus on the improvement of processability along with thermal resistance, mechanical strength, and rheological parameters, in comparison with conventional polymers of petrochemical origin [7,8].

Biopolymer composites are intended to address some of the shortcomings of polymer properties, such as poor mechanical performance, low strength, limited processing capacity, and long-term capability. They are created by reinforcing biopolymers with appropriate fillers, for example, clay minerals [9].

For Carastan [10], clays can be defined as natural and earthy materials that are fine-grained (particles with a diameter generally less than 2 μm) and chemically formed by hydrated silicates of aluminum, iron, and magnesium. They are made up of extremely small crystalline particles of a restricted number of minerals, known as clay minerals. Any clay can be composed of a single clay mineral or a mixture of several of them. Clays may also contain organic matter, soluble salts, quartz particles, pyrite, calcite, other residual minerals, and amorphous minerals. Its structures are dependent upon two basic units, namely an octahedral and a tetrahedral. The tetrahedral structure consists of the presence of silicon (Si4+), as a coordinated central atom, covalently with four oxygen atoms located at the vertices of the tetrahedron. In the absence of substitution atoms or adsorbed ions, the faces of these layers are electrically neutral and stacked up by Van der Waal’s forces, leaving between them a space known as the interlamellar or gallery space. The galleries are then occupied by inorganic cations that counterbalance these negative charges. These cations, once hydrated, can be exchanged for organic salts, such as quaternary ammonium salt, which makes the surface of the galleries organophilic, allowing for the intercalation of monomer or polymer molecules. It is necessary to understand and control the physicochemical interactions between the ion intercalated in the silicate layers and the polymer to be intercalated in the nanoparticles in order to obtain nanocomposites. In general, in the preparation of the nanocomposites, a compatibilizing agent, such as polyethylene grafted with maleic anhydride (PE-g-MA), has been used. The idea is to improve the clay–polymer matrix adhesion. The nanocomposites containing the compatibilizing agent (PE-g-MA) promote improvements in thermal stability when compared to the B-HDPE matrix and form exfoliated nanocomposites.

It is known that composites are used today in diverse sectors, such as biomedicine, civil construction, and aerospace. Silicate-reinforced polymer composites are commonly used in a variety of applications, such as automotive and aerospace, sports, medical implants and drug administration, textiles, packaging, infrastructure and construction, upholstery and furniture, and many household products. The challenge of clay-based composites involves several issues, both in formulation and application, especially regarding material compatibility, processing method, and sustainability. In this context, using green polyethylene to produce composites based on natural fillers becomes essential for the industrial sector, which can boost applications in the automotive sector.

Thus, based on the information already mentioned and the importance of studying biocomposites, the present work aimed to develop and evaluate biocomposites using a sustainable matrix of “green” bio-high-density polyethylene (B-HDPE) and organophilic montmorillonite clay (OMMT) as reinforcement. The idea is to show that the presence of OMMT in the polymer matrix improves the properties of the B-HDPE biocomposite, along with PE-g-MA. The biocomposites were investigated by testing their structural (FTIR and XRD), thermal (DSC and TG), thermomechanical (DMA), morphological (MEV and MET), and mechanical (tensile, flexure, impact, and hardness) properties. The innovative aspect of the research is related to the use of a new reinforcement to produce polymer composites, the organophilic montmorillonite clay.

## 2. Materials and Methods

### 2.1. Materials

The following materials were used in this research.

(a) High-density green polyethylene, SGE7252 trade name (BPEAD), I’m Green^®^, was obtained from sugarcane ethanol and has the positive effect of reducing CO_2_ emissions into the atmosphere. It was supplied by Braskem company (Maceió, Brazil) and had a relative density of 0.952 g/cm^3^ and a melt flow index of IF = 2.0 g/10 min^−1^ [11];
(b) The clay (trademark Dellite 72T) used in this research is derived from natural montmorillonite, specially purified and modified with quaternary ammonium salt (dihydrogenated with dimethyl ammonium) (2M2HT). It is an additive for polymer applications and is used to improve various physical and thermomechanical properties. The average particle size is 7–9 μm, which is the average size after 1 × 500 nm dispersion. The specific weight is 1.7 g/cm^3^, and the bulk density is 0.45 g/cm^3^.

### 2.2. Preparation of Organophilic Montmorillonite Clay (OMMT)

Sodium bentonite clay was purified and organically modified through an ion exchange reaction in an aqueous medium with quaternary ammonium salt (dihydrogenated with dimethyl ammonium) (2M2HT).

### 2.3. Preparation of Biocomposites

**Figure 1:**
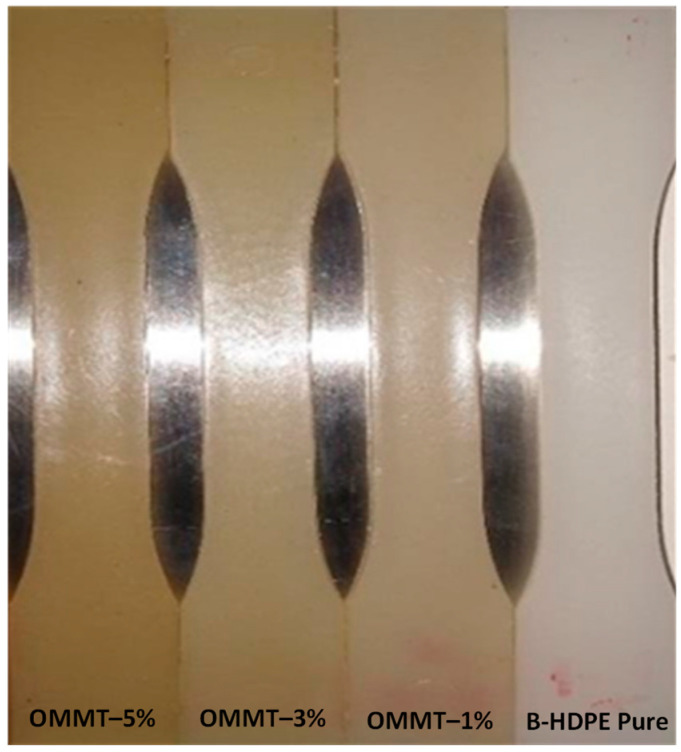
Samples of the biocomposites BPEAD/PE-g- AM/OMMT (1, 3, and 5% OMMT) and BHDPE pure.

The biocomposites were obtained by mixing green polyethylene (B-HDPE) and organophilic montmorillonite clay (OMMT). Further, 10% by weight of the polyethylene grafted with maleic anhydride (PE-g-MA) was added to all of the biocomposites. Initially, the biocomposites were prepared by the molten state method and processed in a co-rotational modular extruder, model LSM 30.34 (LEISTRITZ Manufacturer, Cleveland, OH, USA), screw diameter 30 mm and L/D = 29. The process conditions were a barrel temperature ranging from 160 to 180 °C, a 150 rpm screw speed, and a 2 kg/h feed rate. The BPEAD and the biocomposites were then molded by injection molding in an ARBURG injector from Goldem equipment (Lossburg, Germany), with Tmold = 25 °C, Tzones ranged from 180 to 200 °C, and mold cooling time 5 s. The biocomposites were processed in OMMT proportions of 1, 3, and 5% by weight. After this step, the samples were characterized (Figure 1). Table 1 shows the conditions of the samples evaluated in this study. 

### 2.4. Characterization

#### 2.4.1. X-Ray Diffraction (XRD)

The effect of the concentration of B-HDPE/OMMT compositions on the crystallinity of pure biopolymers was evaluated by X-ray diffraction (XRD). A RAGAKU X-ray diffractometer, MINI FLEX 2 (Wilmington, DE, USA) (Cu Kα radiation), operating in the 2Ɵ range from 2 to 60 degrees, 30 kV, and 15 mA (0.02 min^−1^), was used.

#### 2.4.2. Thermal Analysis by Differential Scanning Calorimetry (DSC)

DSC analyses were performed, in order to identify the melting temperature and the degree of crystallinity of the biocomposites. They were carried out using a SHIMADZU DSC, 50 devices (Kyoto, Japan), under the following operating conditions: heating of the room temperature to 200 °C at a rate of 10 °C/min under a nitrogen (N_2_) atmosphere. To calculate the percentage of crystallinity, the ratio between the variations of the melting enthalpies of the sample (ΔH_ms_) and the enthalpy of 100% crystalline HDPE (ΔH_100%_ = 293 J/g) [12,13] was used, as shown in Equation (1). In this calculation, it is considered that ΔH_ms_ is the same for HDPE and BHDPE since the difference between the two polymers consists only of the route used to prepare the monomer (ethylene).

(1) $$\chi_{c}\left(\%\right)=\left(\frac{{∆H}_{ms}}{{∆H}_{100\%}}\right)\times 100,$$

#### 2.4.3. Thermogravimetry (TG and DTG)

The analyses were carried out on a simultaneous TG/DTG Shimatzu DTG 60H equipment (Kyoto, Japan), using approximately 5 ± 0.5 mg of sample, a heating rate of 10 °C/min, and a gas volumetric flow rate of 50 mL/min. The test started from room temperature to 800 °C in a nitrogen atmosphere.

#### 2.4.4. Thermodynamic–Mechanical Analysis (DMA)

Dynamic thermomechanical tests of the B-HDPE/PE-g-MA/OMMT biocomposites were carried out with DMA equipment, model Q800, TA instruments (New Castle, DE, USA), in bending mode using an oscillation amplitude 20 µm, a frequency of 1 Hz, a heating rate of 2 °C/min, and a temperature range from—50 to 100 °C. The specimen’s dimensions are as follows: 57.5 × 12.73 × 3.12 mm.

#### 2.4.5. Transmission Electron Microscopy (TEM) Analysis

The morphology of the biocomposites was analyzed by the observing images obtained under a transmission electron microscope, model MORGANI, 268 D, FEI Company (Hillsboro, OR, USA). The samples were submitted to trimming and cryogenically microtomized, i.e., cut into ultrathin sections, 40 nm thick, with diamond knives, by means of a Riechert-Jung microtome type Ultracut E, with a sample temperature between −65 and −75 °C and the knife temperature at −50 °C.

#### 2.4.6. Scanning Electron Microscopy (SEM)

The morphologies of the fracture surface of the biocomposites were investigated using a Shimadzu SSX-550 Superscan scanning electron microscope at an accelerating voltage of 20 kV in a high vacuum. The fracture surfaces of the B-HDPE and the biocomposites were obtained after the IZOD impact test and were coated with gold for approximately 40 min.

#### 2.4.7. Mechanical Properties of Biocomposites

The mechanical characterizations were performed using injected specimens. The tensile and flexural tests were performed on a SHIMADZU machine, model AG-X (Kyoto, Japan), with a 10 kN load cell. The tensile test was performed at a speed of 50 mm/min according to ASTM D638 [14]. For bending, the test speed was 2.0 mm/min according to ASTM D790 [15]. The IZOD impact strength test was performed according to the ASTM D256 [16] using CEAST RESIL 5.5 equipment (Charlotte, NC, USA), with a 2.75 J impactor, (the 2.5 mm notches were produced in a CEAST NOTSCHVIS slot machine). The shore D hardness test was performed according to the ASTM D2240 [17], with a fixed load of 50 N. For each characterization, 5 specimens per sample were used.

## 3. Results and Discussions

### 3.1. X-Ray Diffraction (XRD)

**Figure 2:**
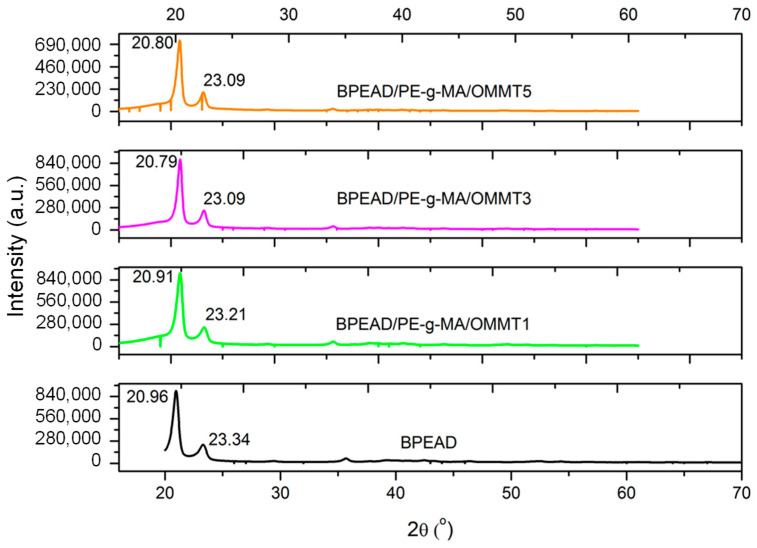
XRD patterns of B-HDPE and biocomposites.

From the XRD analysis, it is evaluated whether there were changes in the positions of the peaks, as well as in their width and height, in order to investigate changes in the crystal structure of the B-HDPE with the incorporation of the OMMT. Figure 2 shows the X-ray diffractograms in the high-angle region for the B-HDPE and OMMT biocomposites. The B-HDPE and the B-HDPE/PE-g-MA/OMMT composites presented the behavior of semicrystalline materials with intense and broad peaks. The 2θ peaks were observed at 20.8° and 23.09° due to the (110) and (200) planes, respectively. This suggests the orthorhombic crystalline structure of polyethylene, as reported in the literature [18]. Regardless of the clay content, the B-HDPE/PE-g-MA/OMMT composites maintained the XRD patterns at 20.8° and 23.09° in relation to the B-HDPE. This was confirmed later in the DSC, given that the crystal melting temperature maintained the same melting pattern in the curves, which suggests an equivalent crystal structure [19].

### 3.2. Thermal Analysis by Differential Scanning Calorimetry (DSC)

**Figure 3:**
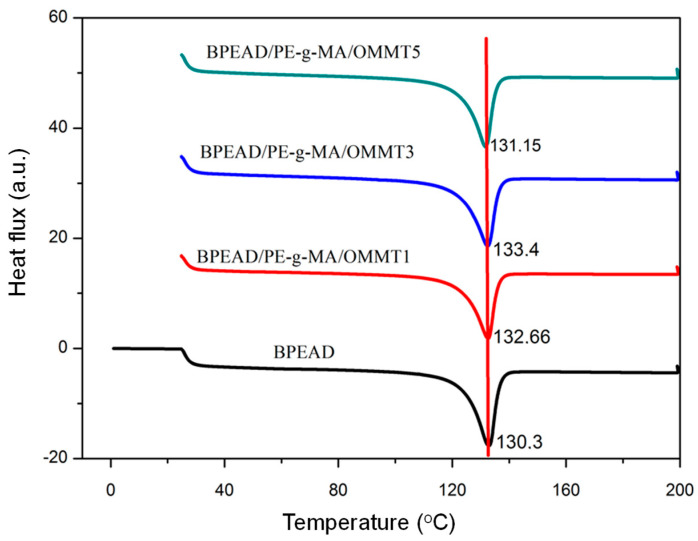
Melting temperature of B-HDPE and OMMT biocomposites.

Figure 3 shows the crystal melting temperature behavior for B-HDPE and B-HDPE/PE-g-MA/OMMT composites with different clay contents. Table 2 shows the values of the melting temperature (T_m_), melting enthalpy (ΔH_m_), and degree of crystallinity (χ_c_) of the B-HDPE and the biocomposites.

The crystal melting temperature (T_m_) of the B-HDPE was 130.3 °C, corroborating the literature [20]. The Tm values of the B-HDPE/PE-g-MA/OMMT composites are very close to those of the B-HDPE, but with a slight increase, as shown in Table 2. Although not a significant difference, the increase in Tm of the composites suggests a slight increase in crystal perfection. This is justified by the various nucleation sites of the clay, where relatively larger crystals are favored. Regarding the enthalpy of crystalline melting (ΔH_m_), B-HDPE required greater energy to melt the crystals. In contrast, the B-HDPE/PE-g-MA/OMMT composites tended to require less energy to melt the crystalline fraction due to the lower crystallinity. In the composites modified by OMMT, the degree of crystallinity (Xc) decreased compared to B-HDPE, which is consistent with what was observed in the literature [21].

The dispersed silicate layers decreased the overall crystallization rate of B-HDPE with a further increase in the OMMT content. At the highest OMMT concentration (5%), the silicate layers behaved as heterogeneities that retard the crystal growth of B-HDPE, as reported in the literature [22]. For the investigated clay contents (1–5%) in the composition, no significant change in the degree of crystallinity was observed between the composites. Furthermore, the Tm curve profile was similar for all samples, suggesting that the crystalline fraction remained in the composites, confirming the XRD trend, as previously verified.

### 3.3. Thermogravimetry

The TG and DTG analyses of the B-HDPE and OMMT biocomposites were evaluated. The thermal degradation behavior of the samples is presented in just one step, which is characteristic of the polymer. The first mass loss, due to B-HDPE degradation, began at the initial degradation temperature (T_onset_), at approximately 393.59 °C, and increased with the addition of 3 and 5% OMMT. These composites begin to degrade at 415.50 and 448.82 °C, with mass losses of about 89.30% and 84.12%, respectively. The B-HDPE/PE-g-MA/OMMT1 biocomposite showed the lowest T_onset_ (349.40 °C), reflecting a decrease of 44.19 °C in T_onset_ in relation to the polymeric matrix (B-HDPE). When 3 and 5% OMMT were present, the clay particles acted as a thermal insulation barrier, which also minimized the permeability of volatile degradation products to the biopolymer (B-HDPE) [23,24]. For biocomposites with 5% by mass (B-HDPE/PE-g-MA/OMMT5), whose T_onset_ of degradation reaches 448.82 °C, there was verified a decrease of 33.32 °C in the T_onset_ in relation to the T_onset_ of the biocomposite (B-HDPE/PE-g-MA/OMMT3). This result suggests the higher content of nanoparticles to be effective as thermal protection for the polymer.

According to the TG curves of the OMMT biocomposites, it is observed that there was a temperature increase in the biocomposites observed at 80% mass loss, T(80), as the final degradation temperature (Tfinal) increased. Although the increases presented few differences among the biocomposites, OMMT 1% by mass presented the lowest thermal resistance, as shown in Table 3, which implies that this lower concentration was insufficient to improve the thermal degradation of the polymer.

### 3.4. Transmission Electron Microscopy (TEM)

**Figure 4:**
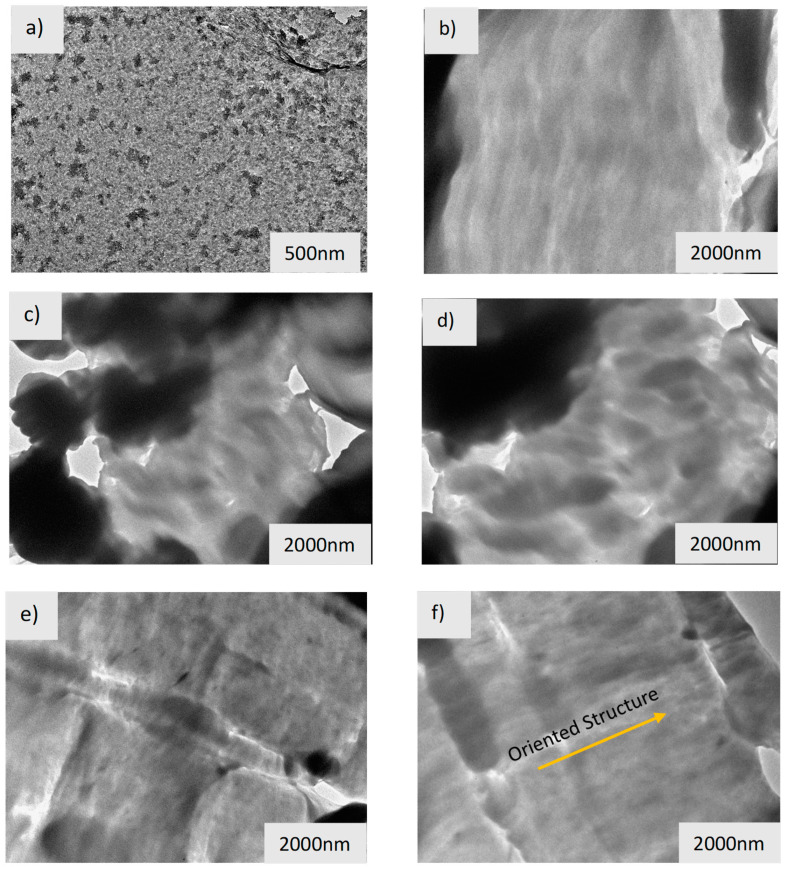
TEM images of B-HDPE-based biocomposites with montmorillonite clay (OMMT). (**a**,**b**) 1% by weight of OMMT, (**c**,**d**) 3% by weight of OMMT, and (**e**,**f**) 5% by weight of OMMT.

Figure 4 shows the degree of intercalation/exfoliation of 1, 3, and 5% by weight of clay (OMMT), with 10% of PE-g-MA, as a compatibilizer in the B-HDPE matrix. The intercalation and/or exfoliation process occurs, as there is diffusion of the macromolecules of the compatibilizing agent or the polymeric matrix in the interlamellar spaces. This process will depend on electrostatic factors (clay/polymer relative polarity) and steric (entropic factors related to the conformation of the polymer chain segments and, consequently, the molecular volume relative to the interlamellar space). On the other hand, the viscosity of the medium also plays an important role in the mobility of the molecules, so that diffusion occurs during the mixing time, and thus, hypothetically, it is expected that polymers with higher viscosity, such as B-HDPE, have favored their diffusion in the interlamellar spaces. For Okamoto et al. [25], in addition, it is important to emphasize that the intercalation process occurs preferentially in the amorphous phase. Thus, the reduction in crystallinity should favor the intercalation and/or exfoliation process, caused by the presence of the compatibilizer (PE-g-MA) used in the formation of biocomposites.

The results are presented in the TEM micrographs, Figure 4c,d, as well as where the clay is well dispersed throughout the matrix, and it is possible to observe the presence of the interspersed structure, as reported in the literature [25]. In these figures, the biocomposites (B-HDPE/PE-g-MA/OMMT3) with 3% OMMT have a lower degree of crystallinity, presenting a good dispersion in a more homogeneous way in the B-HDPE matrix in relation to the biocomposites with 1 and 5% by weight of OMMT. The darker phase represents the inorganic phase of the OMMT, which has regions with many clusters, as shown in Figure 4e,f, although some interspersed layers have been observed. For the biocomposites studied, the use of the compatibilizing agent grafted with maleic anhydride (PE-g-MA), which is miscible with the constituents of the matrix, and helped to obtain a distribution of polar groups by the polymeric matrix, facilitating the dispersion of the clay throughout the matrix.

The results presented in this item corroborate the results obtained by X-ray diffraction and highlight the importance of studying the mixing sequence in the search for greater interactions between the organic matrix and the inorganic load. For Si and Mangal [26], other factors are very important for the production of interspersed and/or exfoliated polyethylene–clay biocomposites, such as the influence of shear on obtaining biocomposites and the compatibility of the polymeric matrix and the inorganic load. Shear forces facilitate the breakdown of large agglomerates, while the extent of clay exfoliation (OMMT) is determined more by the compatibility between the polymer matrix and the clay layers than by the shear forces.

### 3.5. Mechanical Thermodynamic Analysis (DMA)

**Figure 5:**
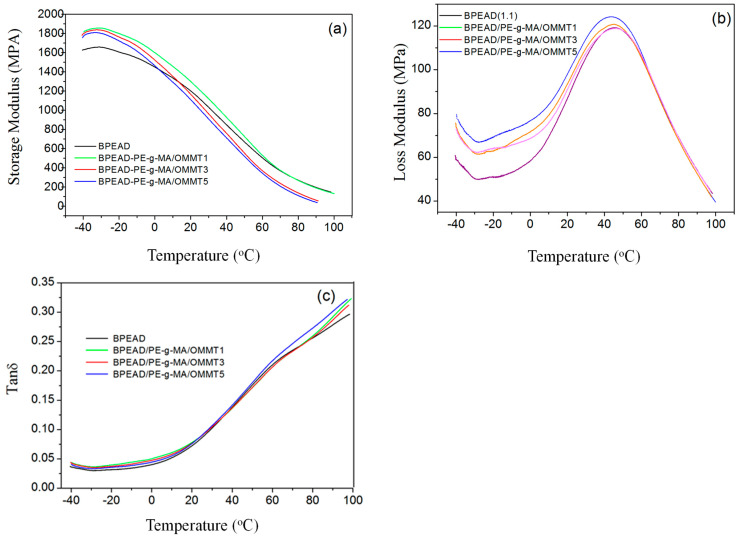
Typical DMA analysis curves: (**a**) storage modulus, (**b**) loss modulus, and (**c**) tan δ.

Dynamic mechanical analysis (DMA) is a very sensitive technique that allows for the determination of the viscoelastic behavior of polymers and provides valuable information about the relationship between the structure, morphology, and properties of biocomposite materials [27]. Dynamic mechanical properties (storage module E′, loss module E″, and tan δ), considering polymeric composites (Figure 5), are sensitive to the amount of fillers and the presence of additives, such as compatibilizers, fiber orientation, clay contents, and interfacial alteration, due to adhesion [28]. The storage module (E′) is related to the material’s ability to resist the loading. The addition of organophilic clay (OMMT) increased E′, mainly for the biocomposites with 3 and 5%, by the mass of the OMMT. It can be observed that, in the temperature range of −40 and 22 °C, there was the greatest variation, with an E′ of around 10% for the biocomposite with 3% and 15% for the biocomposite with 5% by the mass of the OMMT. In the entire temperature range, B-HDPE/PE-g-AM/OMMT5 has a higher E′ compared to B-HDPE and other biocomposites. However, the composites presented similar results in the storage modulus, confirming the trend of similar values of the DSC degree of crystallinity. Relaxation (α) is associated with the mobility of long-chain segments in the crystalline phase. From the E″ curves, the B-HDPE and the biocomposites show a relaxation of around 44 °C, which is in agreement with the research reported in the literature [29], and a relaxation was observed around 35 °C. Tanδ is the ratio of the dissipated energy (E″) per cycle to the stored energy (E′) during the cycle. In the engineering context, a high damping capacity (tanδ) is essential to reduce the effect of vibrations. The tan delta behavior of the biocomposites was increased with the OMMT content. It is interesting to note that the dynamic mechanical properties were not affected by the intercalation state of the OMMT.

### 3.6. Mechanical Properties

**Figure 6:**
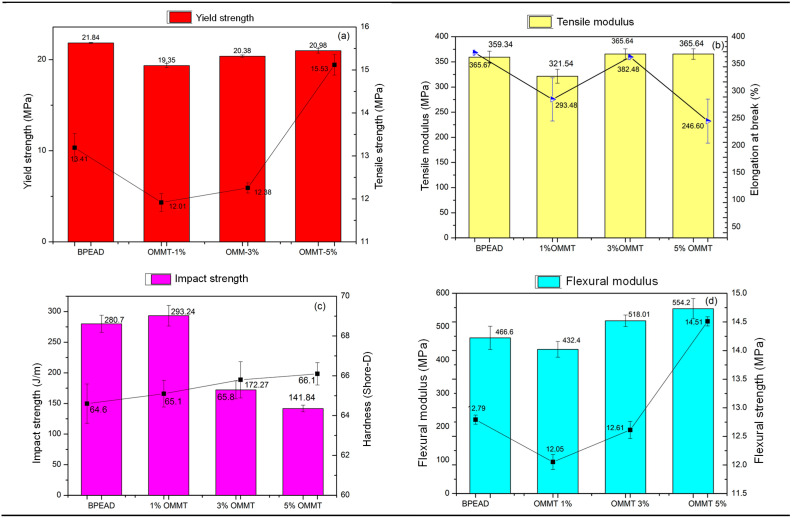
Mechanical properties of BPEAD and OMMT biocomposites: (**a**) yield strength and rupture stress; (**b**) modulus under stress and strain to failure; (**c**) impact resistance and hardness; (**d**) flexural strength and flexural modulus.

Figure 6 presents the comparative graphs of the mechanical properties obtained for tensile, flexure, impact strength, and hardness of BPEAD and OMMT biocomposites. For better understanding, flexural strength is a mechanical property of the material that can be defined as the tensile immediately before it yields in a flexural test. The flexural modulus represents the tendency of a material to resist flexural. Tensile strength corresponds to the maximum stress that a material can withstand when it is axially deformed before failure. The tensile modulus corresponds to the mechanical property that is related to the stiffness (tensile or compression) of the solid material.

From Figure 6a, the yield stress values of the biocomposites remain practically constant, with very little variation in relation to the B-HDPE. Studies developed by Liu and Tu [30] came to the conclusion that the addition of OMMT alters the chemical structure of the polymeric matrix, making it denser and more compact, and it is possible to deduce that the biocomposites have a higher density. For Sarantópoulos et al. [31], all high-density polyethylene has a crystalline and amorphous fraction, where the crystalline fraction is responsible for strength and the amorphous fraction for elasticity, softness, and flexibility. This information may explain the behavior of B-HDPE/PE-g-MA/OMMT biocomposites with 3 and 5 %, which increase their rigidity due to the linearity of the chains. Consequently, the higher density of the BPEAD makes the orientation, alignment, and packaging of the chains more efficient, and the intermolecular forces (Van der Waals) can act more intensely. In Figure 6a, it was observed that the tensile strength of the B-HDPE was on the order of 13.41 MPa. Incorporating 1% and 3% MMT in the B-HDPE matrix reduced the tensile strength by 10.4% and 7.6%, respectively. This indicates no effect of the reinforcing filler at these contents of 1% and 3%, considering the decline in tensile strength. However, when the concentration increased to 5% MMT, the tensile strength increased by 15.8% compared to B-HDPE. At this concentration, the MMT clay acted as a reinforcing filler, suggesting better stress transfer. Apparently, 3% MMT was a critical concentration to promote gains in the tensile strength of B-HDPE, possibly due to the improved interactions between the maleic anhydride of the compatibilizer and the clay layers, as previously discussed.

The tensile strength at break of the biocomposites with 5% by mass was 15.53 MPa against 13.41 MPa for BPEAD, being increased by around 13.6%. The higher OMMT content may have facilitated macromolecular alignment in the direction of the applied force, as reported by [32]. Studies that corroborate the results found in this research were developed by Liu and Tu [30]. These authors show that, when 5% by weight of OMMT was added, the aggregation phenomenon occurred, leading these biocomposites to obtain soft and brittle properties, with an increase in their hardness value and a reduction in their elongation, resulting in less energy absorbed by elastic deformation. Elongation at break for a biocomposite with 5% has the largest reduction of 32% compared to BREAD (Figure 4b), while the hardness value increased by about 2.5% compared to the matrix (Figure 4c). This composite performs the soft and brittle behavior proposed by [33]. The 1 and 3% composites presented an elongation at break and hardness values close to those of the polymer.

The tensile modulus (Figure 6b) was reduced, mainly with the addition of 1% OMMT, around 12%. In relation to biocomposites, with the addition of 3 and 5%, there was an increase of 2.0% in relation to B-HDPE, indicating that biocomposites with OMMT were more rigid than pure polymer, due to the effect of clay reinforcement on the polymer matrix [34,35], while the opposite trend was recorded for the impact strength values, according to the study already published [32].

**Figure 7:**
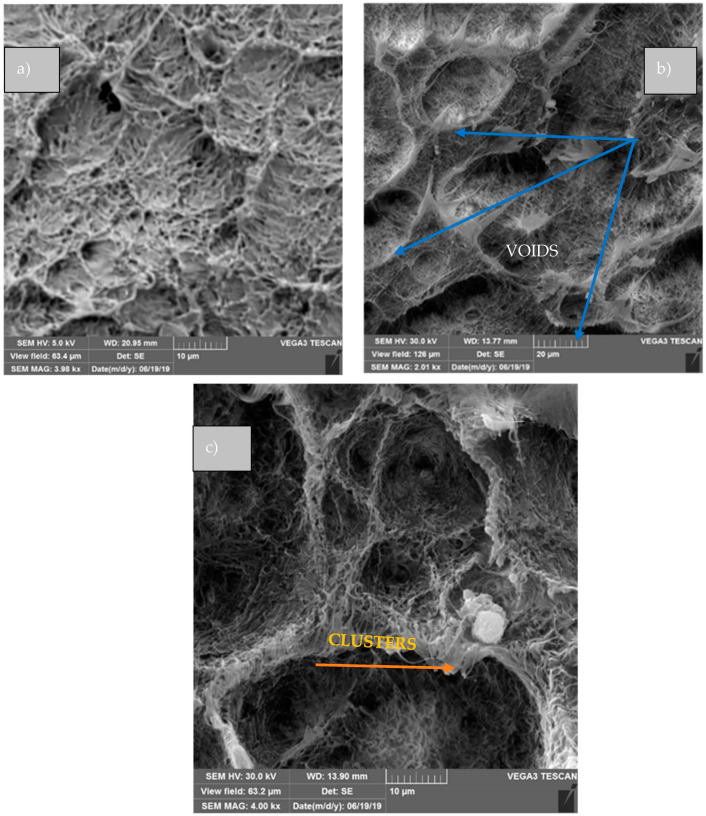
MEV of B-HDPE and OMMT biocomposites: (**a**) 1% OMMT; (**b**) 3% OMMT; and (**c**) 5% OMMT.

Regarding impact resistance (Figure 6c), the addition of 1% OMMT does not decrease this property of the polymer. On the other hand, the 3 and 5% biocomposites present a compromised impact resistance in relation to B-HDPE, where it is more noticeable at 5% in mass, with an intense drop of 49.46%. Fracture surface analyses of the biocomposites shed light on understanding the impact strength of the composites. From the fracture surface of 1% OMMT (Figure 7a), it is seen that the nanoparticles are well dispersed into the polymer, which corroborates with a better response to impact. However, 3 and 5% OMMT, Figure 7b and Figure 7c, respectively, show biocomposites with agglomerates of OMMT and large voids, mainly when higher content was added. These morphological aspects led to heterogeneous surfaces that were not able to resist impact well. The rigidity modulus of these composites was higher than that of the matrix, while under impact, the worst results were observed according to [36].

Incorporating 1% OMMT in the BPEAD/PE-g-MA system did not change the impact strength compared to B-HDPE, only maintaining it within the margin of experimental error. However, a sudden reduction in impact strength values was observed for composites with higher OMMT contents (Figure 6c), which is more pronounced for those with 5% by mass, a decline of 49.46% compared to that obtained for BPEAD. This behavior is due to the greater amount of clay dispersed in the B-HDPE matrix, which brittles the material. The fillers act as stress concentrations, restricting the mobility of the B-HDPE matrix and reducing plastic deformation [37,38,39]. Shore D hardness evaluates the surface resistance to drilling. Figure 6c shows that the hardness of the B-HDPE was around 64.6. Biocomposites with 1 and 3% clay presented values very close to those of the pure polymer. B-HDPE/PE-g-MA/OMMT5 had an increase of 2.5% in relation to the matrix. A good dispersion of the reinforcement within the matrix with stronger interfacial adhesion leads to an increase in the hardness of the composites [40]. The shore D hardness of the biocomposites was slightly higher than that of the B-HDPE.

Regarding the modulus under flexure, Figure 6d shows that the addition of 3% by mass of OMMT also had a positive effect on this property, with an increase of 10.10%, while the biocomposite with 5% by mass had an increase of 19.15% in relation to B-HDPE. From a 3% mass of organophilic montmorillonite clay (OMMT), the reinforcing capacity of the biocomposite was also observed under bending stress. The mechanical response under flexion of the biocomposites was superior when compared to the tensile strength. These results are in line with other results reported in the literature [41], which examined the behavior of polypropylene nanocomposites prepared to organophilic clay. These authors found that composites containing 2.5% by weight of clay exhibited a 23% higher flexural modulus than pure PP. For a similar report, we can also cite reference [42], which examined the behavior of sol–gel silica–montmorillonite nanoclay composites.

Mechanical properties under tension and bending are correlated with the results of DMA and thermal analysis. In general, the addition of 1% OMMT has no positive effect on the polymer properties. From 3% OMMT onwards, a more effective improvement in these properties begins to be observed. From the DSC data, the 5% OMMT composite showed a slightly better degree of crystallinity than the others. This contributes to better results since, also, an oriented structure of the matrix can be observed in detail in Figure 4f. Impact resistance is not a property of interest for biocomposites with 3 and 5% OMMT. While 1% OMMT showed impact resistance close to that of the matrix, this biocomposite is only suitable for applications involving dynamic mechanical and impact stresses.

### 3.7. Final Comments

As already mentioned, in this work, different analyses were carried out with the aim of evaluating the mechanical performance of biocomposite materials.

In general, the biocomposites were more temperature resistant than the pure polymer, bringing an increase of 21 °C to the maximum degradation temperature with the addition of 5% by weight of OMMT. After the impact test, the fracture surface (evaluated by TEM) revealed that the addition of clay particles into the pure polymer showed a greater alteration of the surface structure with the presence of good adhesion for 1, 3, and 5% OMMT fractions. There was great dispersion of the clay throughout the matrix, and the presence of the intercalated structure proved that some factors are very important for the production of interspersed and/or exfoliated polyethylene–clay biocomposites, such as the influence of shear in obtaining biocomposites and the compatibility of the polymeric matrix and the inorganic load. Similar results have been reported by Meera et al. [42] in their study about sol–gel silica–montmorillonite nanoclay composites.

The shear forces facilitate the breakdown of the large agglomerates, while the extent of clay exfoliation (OMMT) is determined more by the compatibility between the polymer matrix and the clay layers than by the shear forces producing biocomposites with better mechanical and thermal properties. The interfacial adhesion between the reinforcement and the matrix plays an important role and, in fact, influences the physical and mechanical properties of OMMT biocomposites.

DMA analysis suggested interfacial interaction between B-HDPE and OMMT due to an increase in storage module E′ and a decrease in tan δ, especially for biocomposites with 1% by weight of OMMT. The mechanical properties under stress indicated that the tensile strength of B-HDPE remained essentially unchanged. While the biocomposite with 5% by weight of OMMT increased by only 1%, the elongation at break was reduced as a consequence of the compatibilization. The modulus of elasticity B-HDPE was reduced. However, for the biocomposite with 1% by weight of OMMT, this parameter was less affected. The impact resistance decreased concomitantly with the increase in hardness. For bending, the results demonstrated increases in both maximum stress and modulus for the biocomposites with reinforcement of 3 and 5% by weight of OMMT.

Finally, the innovation of the present work lies in demonstrating that small amounts of chemically modified clay can present synergistic effects by increasing the matrix properties, such as resistance to thermal degradation, hardness, and mechanical properties under bending and damping characteristics. All these characteristics are of interest for biocomposite applications in various sectors of the automotive and aeronautical industries, for example. Green polymers are gaining prominence in the automotive industry due to the growing demand for sustainability and reduced environmental impacts. However, it is necessary to investigate the properties of producing new materials for the industry in detail. In addition, the use of additives to improve the performance of green polymers must be consistent with the environmental appeal and at a viable cost [43,44].

## 4. Conclusions

In this work, small amounts of organophilic clay (OMMT) and a compatibilizing agent (PE-g-MA) were used to modify the properties of B-HDPE. From the analyses of the results, we conclude that:

(a) DSC reports indicate that the presence of clay reduces the degree of crystallinity of the polymer by about 16%, increasing the temperature resistance compared to the pure polymer;
(b) TEM analysis revealed that the addition of clay particles into the pure polymer modified the surface structure and provoked great dispersion of the particles throughout the matrix;
(c) DMA analysis promoted interfacial interaction between B-HDPE and OMMT, increasing the storage module and decreasing the tan δ, especially for biocomposites with 1% by weight of OMMT;
(d) The mechanical properties under stress indicated that the tensile strength of BPEAD remained essentially unchanged. A biocomposite with 5% by weight of OMMT increased by only 1%. The elongation at break was reduced as a consequence of the compatibilization. The modulus of elasticity B-HDPE was reduced. The impact resistance decreased, and the hardness increased;
(e) The study proved that the economical use of organophilic clay allows for biocomposite processing parameters equivalent to those used for pure polymers, without altering the processability of B-HDPE.

## Figures and Tables

**Table 1:** Sample composition and labeling.

Denomination	OMMT (%)	PE-g-MA (%)
BPEAD	0	0
B-HDPE/PE-g-MA/OMMT1	1	10
B-HDPE/PE-g-MA/OMMT3	3	10
B-HDPE/PE-g-MA/OMMT5	5	10

**Table 2:** DSC results of B-HDPE and biocomposites.

Samples	T_m_ (°C)	ΔH_m_ (J/g)	χ_c_ (%)
B-HDPE	130.30	120.60	41.16
B-HDPE/PE-g-MA/OMMT1	132.75	98.33	33.55
B-HDPE/PE-g-MA/OMMT3	132.32	97.22	33.18
B-HDPE/PE-g-MA/OMMT5	131.29	101.00	34.47

**Table 3:** Initial temperature, temperatures at 80% mass loss, maximum and final temperature of B-HDPE, and its OMMT biocomposites presented in TG and DTG.

Temperature (t)	B-HDPE	BHDPE/PE-g-MA/OMMT1	BHDPE/PE-g-MA/OMMT3	BHDPE/PE-g-MA/OMMT5
T_onset_ (°C)	393.59	349.40	415.50	448.82
T(80) (°C)	406.81	384.27	438.75	467.23
T_max_ (°C)	459.87	415.68	487.08	490.19
T_final_ (°C)	474.60	485.64	493.93	502.56

## Data Availability

The original contributions presented in the study are included in the article, further inquiries can be directed to the corresponding author.
